# Compound screen identifies the small molecule Q34 as an inhibitor of SARS-CoV-2 infection

**DOI:** 10.1016/j.isci.2021.103684

**Published:** 2021-12-24

**Authors:** Qi Cui, Gustavo Garcia, Mingzi Zhang, Cheng Wang, Hongzhi Li, Tao Zhou, Guihua Sun, Vaithilingaraja Arumugaswami, Yanhong Shi

**Affiliations:** 1 Division of Stem Cell Biology Research, Department of Developmental and Stem Cell Biology, Beckman Research Institute of City of Hope, Duarte, CA 91010, USA; 2 Department of Molecular and Medical Pharmacology, University of California, Los Angeles, Los Angeles, CA 90095, USA; 3 Department of Molecular Medicine, Beckman Research Institute of City of Hope, Duarte, CA 91010, USA; 4 Diabetes and Metabolism Research Institute at City of Hope, Duarte, CA 91010, USA; 5 Eli and Edythe Broad Center of Regenerative Medicine and Stem Cell Research, University of California, Los Angeles, Los Angeles, CA 90095, USA; 6 Irell & Manella Graduate School of Biological Sciences, Beckman Research Institute of City of Hope, Duarte, CA 91010, USA

**Keywords:** Chemistry, Small molecule, Virology, Cell biology

## Abstract

The COVID-19 outbreak poses a serious threat to global public health. Effective countermeasures and approved therapeutics are desperately needed. In this study, we screened a small molecule library containing the NCI-DTP compounds to identify molecules that can prevent SARS-CoV-2 cellular entry. By applying a luciferase assay-based screening using a pseudotyped SARS-CoV-2-mediated cell entry assay, we identified a small molecule compound Q34 that can efficiently block cellular entry of the pseudotyped SARS-CoV-2 into human ACE2-expressing HEK293T cells, and inhibit the infection of the authentic SARS-CoV-2 in human ACE2-expressing HEK293T cells, human iPSC-derived neurons and astrocytes, and human lung Calu-3 cells. Importantly, the safety profile of the compound is favorable. There is no obvious toxicity observed in uninfected cells treated with the compound. Thus, this compound holds great potential as both prophylactics and therapeutics for COVID-19 and future pandemics by blocking the entry of SARS-CoV-2 and related viruses into human cells.

## Highlights

• A compound library was screened to identify inhibitors of SARS-CoV-2 cellular entry
• Small molecule Q34 is a potent inhibitor of cellular entry of pseudotyped SARS-CoV-2
• Compound Q34 inhibits authentic SARS-CoV-2 infection of human cells
• Compound Q34 is non-toxic to human cells without SARS-CoV-2 infection

## Graphical abstract

**Figure:**
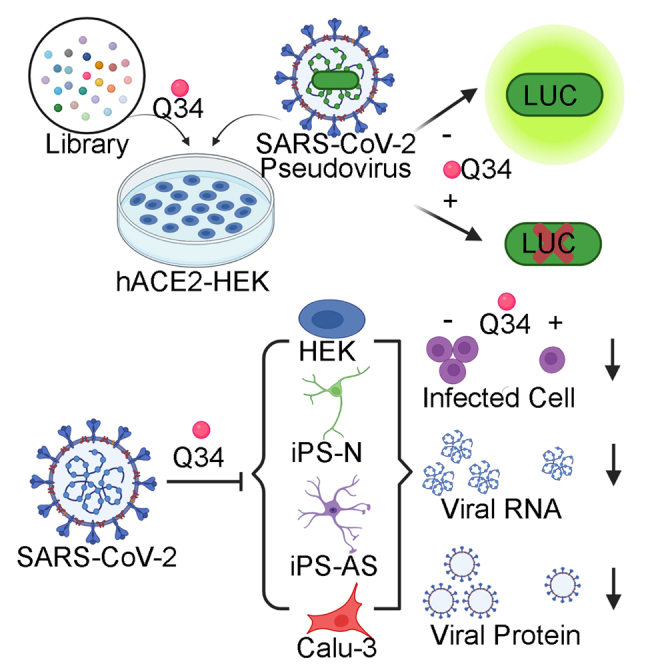

## Introduction

The coronavirus disease 2019 (COVID-19) is caused by severe acute respiratory syndrome coronavirus 2 (SARS-CoV-2) (Zhu et al., 2020; Wu et al., 2020b, Coronaviridae Study Group Of The International Committee On Taxonomy Of, 2020). The rapid and widespread outbreak of SARS-CoV-2 poses a serious threat to global public health. However, there are no approved therapeutics for the treatment or prevention of SARS-CoV-2 infection in clinics. Although a number of nonspecific antiviral drugs including remdesivir and chloroquine have been used in clinics to treat SARS-CoV-2 infection (Wang et al., 2020a; Riva et al., 2020), the *in vivo* efficacy and safety of these drugs remain to be confirmed. Therefore, there is an urgent need for the prompt development of effective therapeutics and prophylactics for the treatment and prevention of COVID-19.

Increased vaccination has reduced SARS-CoV-2 spread substantially. Moreover, the FDA has given remdesivir, an inhibitor of viral RNA polymerase (Warren et al., 2016), emergency use authorization (EUA) for the treatment of COVID-19, because of its effect on reduced time to recover in treated patients in a clinical trial (Team, 2020). Several other existing antiviral therapies, including the HIV-1 protease inhibitors lopinavir/ritonavir, the hepatitis C virus protease inhibitor danoprevir, and the influenza antiviral inhibitor favipiravir, have also been under clinical studies for repurposing to treat COVID-19 (ClinicalTrials.gov). Besides the antiviral therapies that are under clinical investigations, developing novel inhibitors for COVID-19 are needed in order to enhance clinical efficacy and provide more options for combinatorial therapies.

A coronavirus has four structural components, including the spike, envelope, membrane, and nucleocapsid proteins (Wang et al., 2020b; Du et al., 2016; Zhou et al., 2018). Among these components, the spike protein plays the most critical roles in viral attachment and entry into host cells (Du et al., 2009). The entry of coronaviruses into host cells relies on the binding of the spike protein to a cellular receptor and subsequent priming of the spike protein by cellular proteases.

The interaction of the cellular receptor with the virus is an essential aspect that determines the infectivity and host range of coronavirus (Perlman and Netland, 2009; Li, 2016). Angiotensin-converting enzyme-related carboxypeptidase (ACE2) is a protein that is expressed on the surface of the cell membrane. It has been shown that SARS-CoV-2 uses ACE2 as its cellular receptor (Zhou et al., 2020; Letko et al., 2020; Hoffmann et al., 2020; Walls et al., 2020; Shang et al., 2020a). ACE2 is required for host cell entry and subsequent replication of SARS-CoV-2. The spike protein of SARS-CoV-2 binds to ACE2 on the surface of host cells to initiate events that release the viral genome into host cells.

Because the viral entry step serves as an early step during viral life circle, prevention of viral entry can be a strategy to efficiently block viral infection and spread in affected tissue. We propose to identify small molecules that target the viral entry in order to prevent SARS-CoV-2 infection, which could lead to the development of effective prophylactics and therapeutics for COVID-19 and future related pandemics.

## Results

### Identification of compound Q34 as a potent inhibitor of SARS-CoV-2 cellular entry

To identify potential inhibitors for SARS-CoV-2 infection, we performed virtual screening of a small molecule compound library containing >260,000 NCI-DTP (developmental therapeutics program) compounds to identify molecules that are predicted to block the protein–protein interaction between hACE2 and the SARS-CoV-2 spike protein. Among the 209 compounds identified, 118 compounds were readily available. These compounds were obtained and subjected to secondary screening to identify compounds that can interfere with the infection of SARS-CoV-2 pseudovirus using a SARS-CoV-2 pseudovirus system. It has been shown that the pseudovirus system that contains the coronavirus spike proteins could faithfully recapitulate key features of coronavirus entry into host cells (Hoffmann et al., 2020). We used the HIV packaging system (Li et al., 2018b), including plasmids encoding MDL, REV, and the pHIV7-eGFP-ffLuc vector, together with a SARS-CoV-2 spike protein-encoding vector (Shang et al., 2020b) to make the SARS-CoV-2 pseudovirus. The transfer plasmid pHIV7-eGFP-ffLuc contains a luciferase reporter that is used to monitor the cellular entry by the SARS-CoV-2 pseudovirus. Because human ACE2 (hACE2) has been identified as the host receptor for the SARS-CoV-2 spike protein in human cells (Zhou et al., 2020; Letko et al., 2020; Hoffmann et al., 2020; Walls et al., 2020), we transfected hACE2 into human kidney HEK293T cells to make hACE2-HEK293T (hACE2-HEK) and used these cells as host cells for the pseudovirus as we described previously (Sun et al., 2021). The expression of the ACE2 protein was confirmed by immunostaining and Western blot analyses (Figure S1).

**Figure 1:**
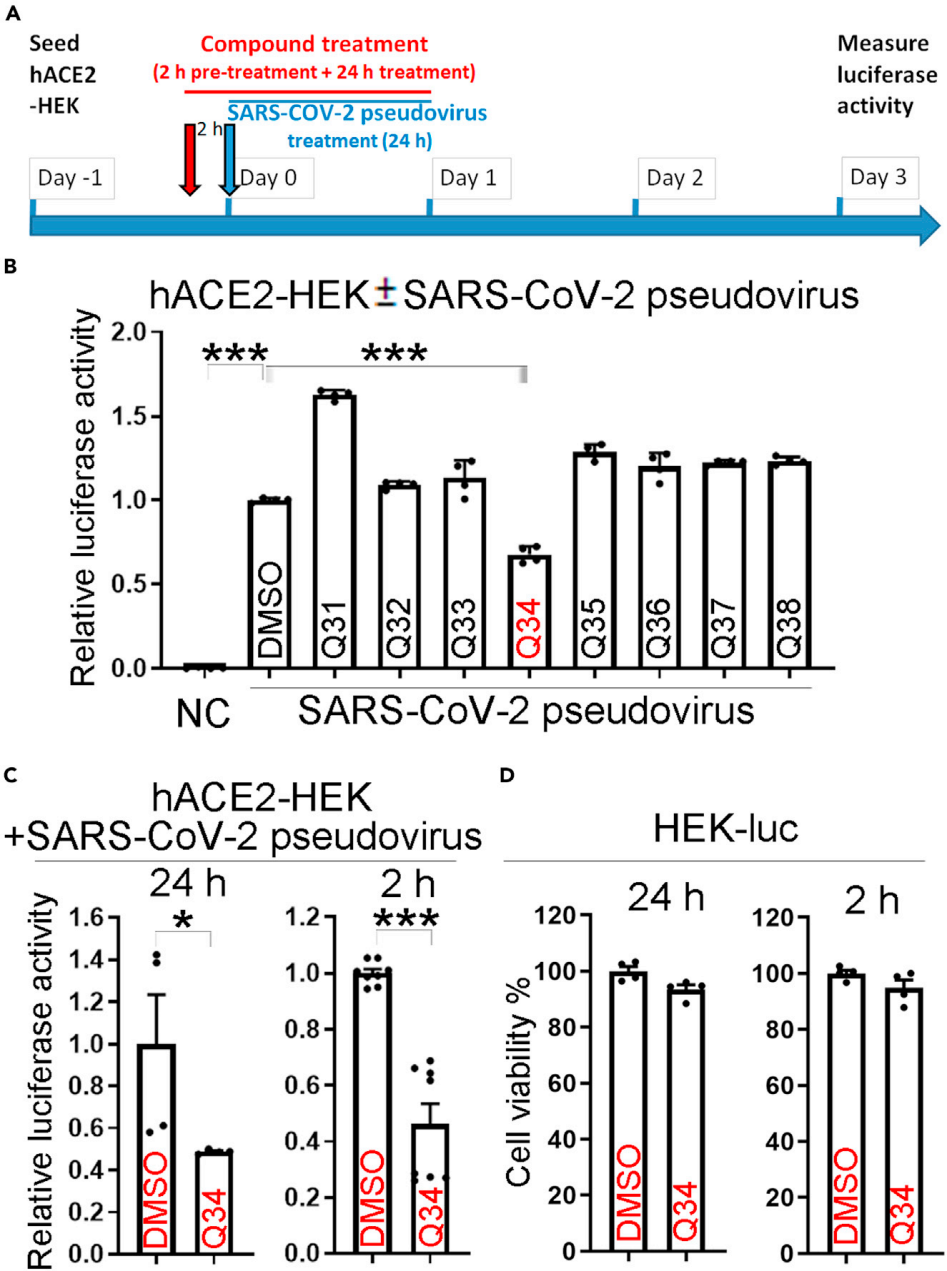
Identification of compound Q34 as an inhibitor of SARS-CoV-2 cellular entry (A) Schematics for screen of candidate inhibitors against pseudotyped SARS-CoV-2 entry into hACE2-HEK cells, including cell seeding and treatment with compounds and SARS-CoV-2 pseudovirus. (B) Compound Q34 inhibits pseudotyped SARS-CoV-2 entry into hACE2-HEK cells. n = 4 experimental replicates. ∗∗∗p < 0.001 by one-way ANOVA test. (C) Validation of compound Q34 in inhibition of cellular entry of pseudotyped SARS-CoV-2. n = 4 experimental replicates (for 24 h time point), n = 8 experimental replicates (for 2 h time point). ∗p < 0.05, and ∗∗∗p < 0.001 by Student’s t test. (D) Toxicity test of compound Q34 in HEK293T-luc (HEK-luc) cells. n = 4 experimental replicates. Error bars are SE of the mean for panels (B), (C) and (D). See also Figure S1.

For compound screening, hACE2-HEK cells were pre-treated with the vehicle control (DMSO) or 10 μM candidate compounds for 2 h, followed by infection with the SARS-CoV-2 pseudovirus along with the compound treatment for 24 h (Figure 1A). While hACE2-HEK cells alone had no detectable luciferase activity, hACE2-HEK cells infected with the SARS-CoV-2 pseudovirus carrying a luciferase reporter and pre-treated with DMSO exhibited potent luciferase activity (Figure 1B). Chloroquine and an anti-spike antibody were used as positive controls for SARS-CoV-2 entry inhibition. Both chloroquine and the anti-spike antibody reduced the luciferase reporter activity in SARS-CoV-2 pseudovirus-treated cells compared to the DMSO or the IgG control, respectively (Figure S1C), validating the capacity of the pseudoviral system for identifying entry inhibitors. When hACE2-HEK cells were pre-treated with the candidate compounds followed by infection with the SARS-CoV-2 pseudovirus, compound Q34 efficiently reduced the luciferase reporter activity in SARS-CoV-2 pseudovirus-treated cells, compared to the DMSO control (Figure 1B). The identifying number assigned by NCI-DTP for compound Q34 is NSC621601. Among the 118 compounds tested, Q34 exhibited the most potent inhibitory effect on SARS-CoV-2 pseudovirus cellular entry. These data indicate that compound Q34 could inhibit the entry of SARS-CoV-2 pseudovirus into host cells.

To validate the screening result, we repeated the experiment by pre-treating the hACE2-HEK cells with 10 μM compound Q34 for 2 h followed by infection with the SARS-CoV-2 pseudovirus along with the compound treatment for 24 h. Q34 reduced luciferase activity from SARS-CoV-2 pseudovirus substantially, compared to the DMSO control (Figure 1C, left panel). To find out if the reduced luciferase activity could be resulted from general cytotoxicity of compound treatment, we performed a control experiment by infection of HEK293T cells with a luciferase reporter-encoding lentivirus to get HEK-luc cells and treated these cells with 10 μM Q34 for 2 h followed for 24 h (2 h + 24 h), mimicking the treatment paradigm in the efficacy test. Treatment with Q34 did not affect the luciferase activity, presented as the percentage of cell viability, from HEK-luc cells, compared to the DMSO control (Figure 1D, left panel), suggesting that Q34 is not toxic to cells not infected by SARS-CoV-2.

In addition to treating cells with Q34 for 2 h pre-infection and 24 h along infection (2 h + 24 h), we tested a shorter treatment paradigm of 2 h pre-infection and 2 h along infection (2 h + 2 h). hACE2-HEK cells were pre-treated with 10 μM Q34 for 2 h, followed by treatment with the SARS-CoV-2 pseudovirus along with the compound for 2 h. Then the pseudovirus and the compound were both removed. DMSO was included as the vehicle control for Q34. Compound Q34 efficiently inhibited the entry of SARS-CoV-2 pseudovirus, compared to the DMSO control, as revealed by substantially reduced luciferase activity from SARS-CoV-2 pseudovirus in Q34-treated cells (Figure 1C, right panel). In contrast, treatment of the control HEK-luc cells with Q34 with the same concentration and for the same treatment period (2 h + 2 h) did not lead to reduced cell viability (Figure 1D, right panel), suggesting that the inhibitory effect of viral infection was not likely resulted from general cytotoxicity of compound treatment. Taken together, these results indicate that compound Q34 is a potent inhibitor of SARS-CoV-2 entry into host cells without general cytotoxicity.

**Figure 2:**
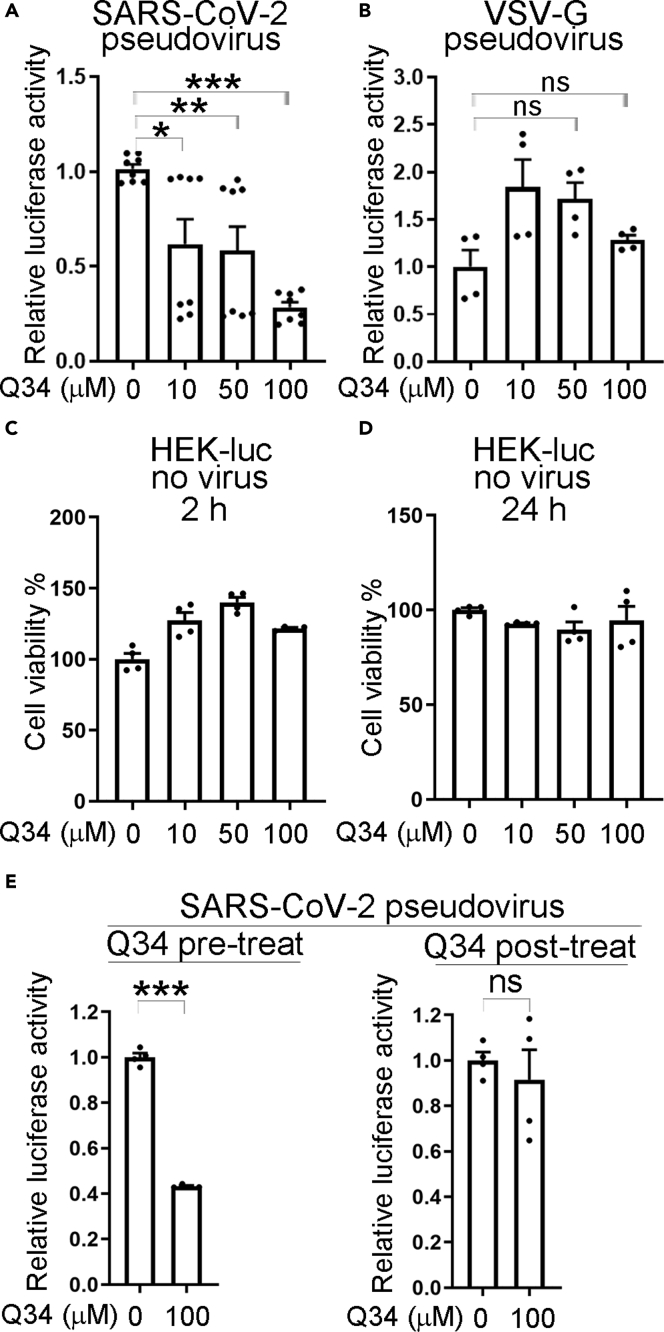
Compound Q34 specifically affects cellular entry of SARS-CoV-2 pseudovirus (A) Dose response of compound Q34 in inhibition of pseudotyped SARS-CoV-2 entry into hACE2-HEK cells. n = 8 experimental replicates. ∗p < 0.05, ∗∗p < 0.01, and ∗∗∗p < 0.001 by one-way ANOVA test. (B) Compound Q34 does not inhibit pseudotyped VSV-G cellular entry. n = 4 experimental replicates. ns means p> 0.05 by one-way ANOVA test. (C) Toxicity test of compound Q34 treatment (2 h pre-treatment plus 2 h treatment) of HEK-luc cells. n = 4 experimental replicates. (D) Toxicity test of compound Q34 treatment (2 h pre-treatment plus 24 h treatment) of HEK-luc cells. n = 4 experimental replicates. (E) Time-of-drug-addition assay to test compound Q34 in inhibition of pseudotyped SARS-CoV-2 entry into hACE2-HEK cells by either pre-treatment (Q34 pre-treatment for 2 h followed by viral treatment for 2 h) or post-treatment (viral treatment for 2 h followed by Q34 post-treatment for 22 h). n = 4 experimental replicates. ∗∗∗p < 0.001 and ns means p> 0.05 by Student’s t test. Error bars are SE of the mean for panels (A–E). See also Figure S2.

To test whether compound Q34 specifically affects cellular entry mediated by the SARS-CoV-2 spike protein, we prepared pseudovirus bearing either the SARS-CoV-2 spike protein or the glycoprotein of the vesicular stomatitis virus (VSV-G). The VSV-G pseudovirus was prepared by transfecting a VSV-G expression vector, instead of the SARS-COV-2 spike protein expression vector, along with MDL, REV, and pHIV7-eGFP-Luc into HEK293T cells for viral packaging. To test the compound effects, hACE2-HEK cells were pre-treated with Q34 at a concentration range of 0–100 μM for 2 h, followed by infection with the SARS-CoV-2 pseudovirus or the VSV-G pseudovirus along with the compound treatment for 2 h. Compound Q34 specifically reduced luciferase activity from the SARS-CoV-2 pseudovirus-treated cells in a dose-dependent manner (Figure 2A), with the strongest inhibitory effect by 100 μM Q34. However, Q34 had no inhibition on luciferase activity in VSV-G pseudovirus-treated cells in all doses tested (Figure 2B). These results indicate that compound Q34 inhibits cellular entry of virus mediated by the SARS-CoV-2 spike protein specifically, but not by the VSV-G glycoprotein.

In contrast, treatment of the control HEK-luc cells with Q34 with the same concentrations and for the same treatment period (2 h + 2 h) did not reduce cell viability (Figure 2C), suggesting that the inhibitory effect of Q34 on SARS-CoV-2 pseudovirus is not likely resulted from general cytotoxicity of compound treatment. The lack of toxicity was revealed by the sustained cell viability in HEK-luc cells even after 2 h + 24 h compound treatment (Figure 2D).

To further test the antiviral activity of compound Q34, a time-of-drug-addition assay was performed. In a pre-treatment condition, hACE2-HEK cells were pre-treated with Q34 for 2 h, then the compound was removed, and the cells were treated with SARS-CoV-2 pseudovirus for 2 h. In a post-treatment condition, hACE2-HEK cells were treated with SARS-CoV-2 pseudovirus for 2 h, then the virus was removed and the cells were treated with Q34 for 22 h. Q34 pre-treatment dramatically inhibited luciferase activity from the SARS-CoV-2 pseudovirus-treated cells, compared to the DMSO control (Figure 2E, left panel). In contrast, there was barely any inhibitory effect on the luciferase activity from SARS-CoV-2 pseudovirus-treated cells when Q34 was added post viral treatment (Figure 2E, right panel), suggesting that Q34 inhibits viral entry.

Because Q34 was predicted to block the interaction between hACE2 and the SARS-CoV-2 spike protein in the initial virtual screen, we tested the effect of Q34 on the interaction of hACE2 with SARS-CoV-2 spike experimentally. To our surprise, we did not observe the disruption of hACE2-spike interaction by Q34 (Figure S2A). Q34 did not inhibit the activity of the endosomal proteases cathepsin B and L either (Figures S2B and S2C), which have been shown to be used for SARS-CoV-2 priming in TMPRSS2-negative cells such as HEK cells (Hoffmann et al., 2020). Moreover, overexpression of TMPRSS2 or the combination of TMPRSS2 and NRP1 in hACE2-HEK cells increased pseudotyped SARS-CoV-2 entry in both DMSO-treated or Q34-treated cells, compared to hACE2-HEK cells (without overexpression of TMPRSS2 and/or NRP1) under the same treatment condition, suggesting that Q34 may not act through TMPRSS2 or NRP1 (Figure S2D). Taken together, these results indicate that compound Q34 inhibits the entry of SARS-CoV-2 spike protein pseudotyped virus.

### Compound Q34 inhibits SARS-CoV-2 cellular entry with a broad safety dose range

**Figure 3:**
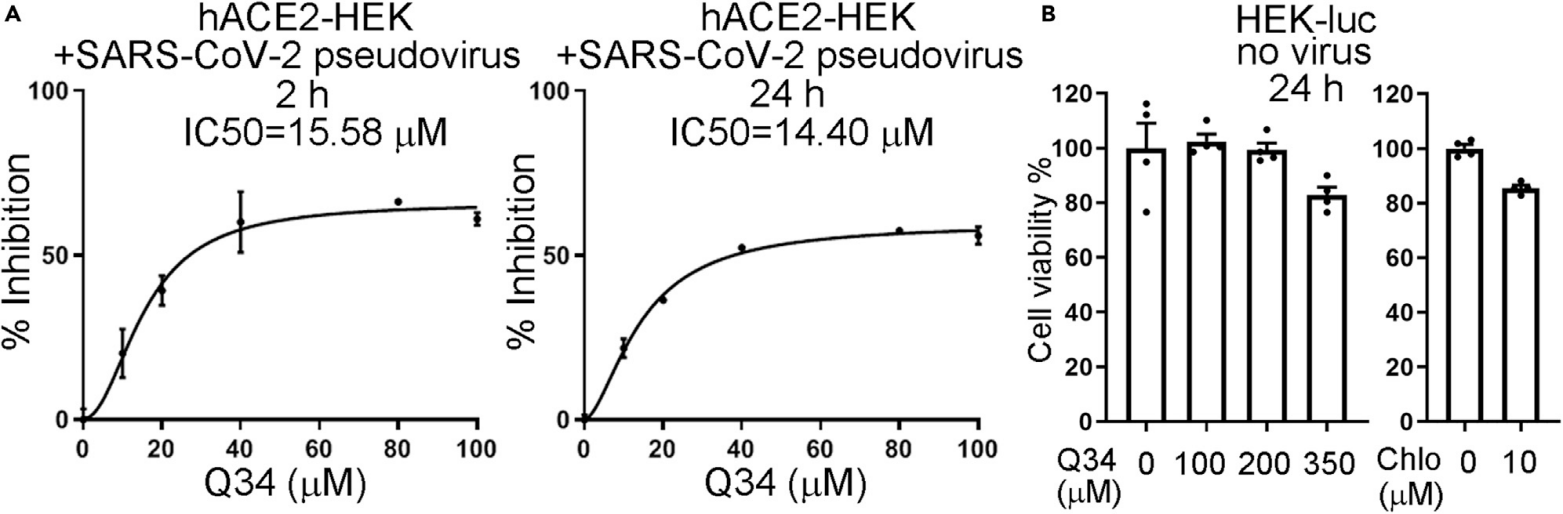
IC50 and safety dose range test of compound Q34 (A) Dose response of compound Q34 in inhibition of pseudotyped SARS-CoV-2 entry into hACE2-HEK cells. n = 4 experimental replicates. Left panel: 2 h pre-treatment plus 2 h treatment; right panel: 2 h pre-treatment plus 24 h treatment. (B) Toxicity test of compound Q34 or chloroquine (Chlo) treatment (2 h pre-treatment plus 24 h treatment) of HEK-luc cells. n = 4 experimental replicates. Error bars are SE of the mean for (A and B).

To test the IC50 dose of Q34 in inhibiting cellular entry mediated by the SARS-CoV-2 spike protein, a range of Q34 compounds (0–100 μM) was tested. hACE2-HEK cells were pre-treated with Q34 at a concentration range of 0–100 μM for 2 h, followed by infection with the SARS-CoV-2 pseudovirus along with the compound treatment for 2 h or 24 h. Q34 inhibited the infection of SARS-CoV-2 pseudovirus in a dose-dependent manner for both treatment periods with an IC50 of 15.58 μM for 2 h + 2 h treatment period and an IC50 of 14.40 μM for 2 h + 24 h period (Figure 3A). In addition, we evaluated a safety dose range for Q34 by treating HEK-luc cells with Q34 at a concentration range of 0–350 μM. No effect on cell viability was observed when HEK-luc cells were treated with Q34 at 200 μM or below, while a mild reduction of cell viability (<20%) was seen when HEK-luc cells were treated with Q34 at 350 μM (Figure 3B). In the same assay system tested for Q34, a mild reduction of cell viability was observed when HEK-luc cells were treated with chloroquine at 10 μM (Figure 3B). Taken together, these data indicate that Q34 inhibits infection of SARS-CoV-2 pseudovirus in a dose-dependent manner and exhibits no cytotoxicity in a broad dose range.

### Compound Q34 inhibits authentic SARS-CoV-2 infection of hACE2-HEK cells

**Figure 4:**
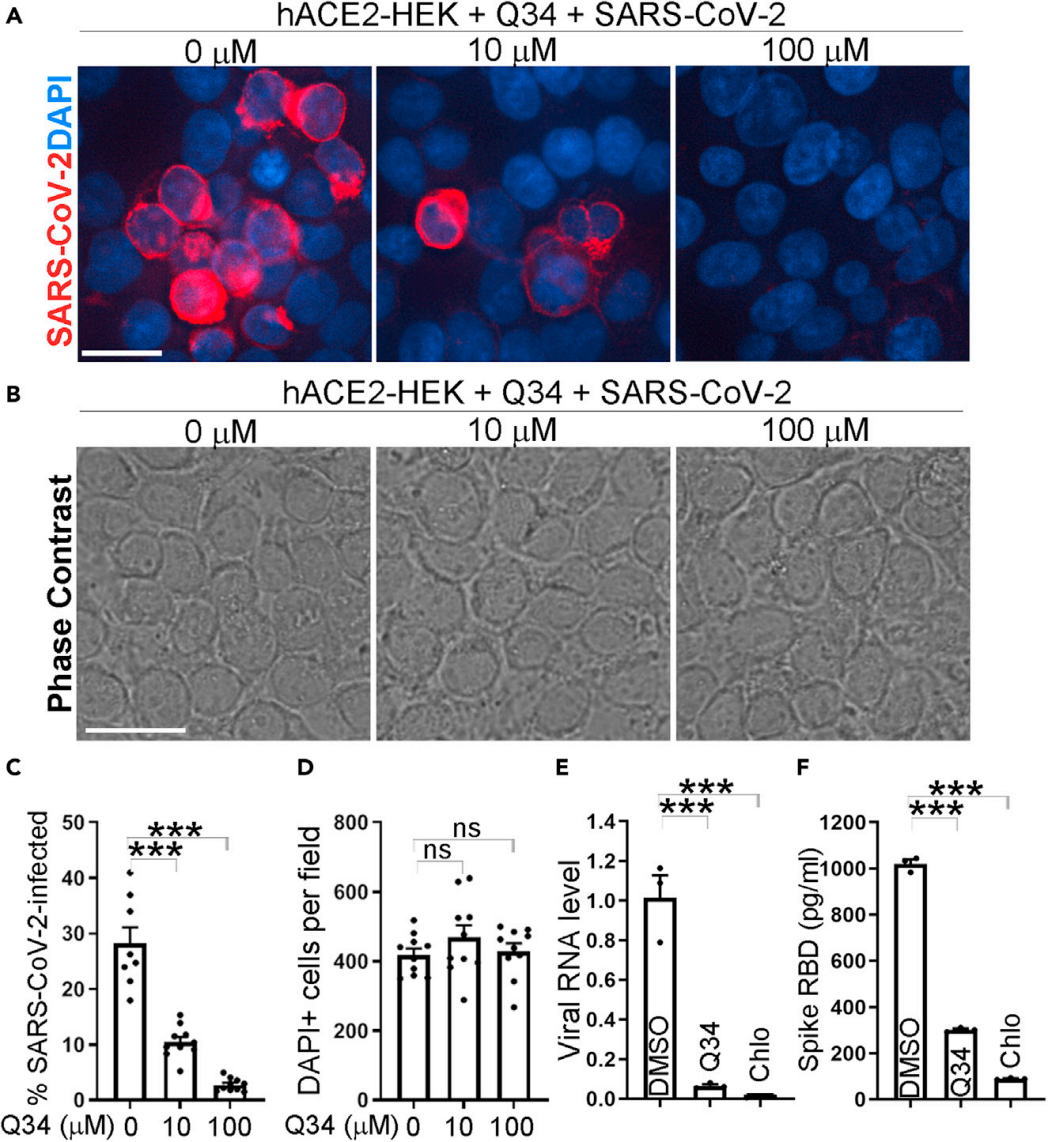
Compound Q34 inhibits SARS-CoV-2 infection (A) Immunostaining for SARS-CoV-2 in hACE2-HEK cells treated with compound Q34 at concentrations from 0 to 100 μM along with SARS-CoV-2 virus. Scale bar: 50 μm. (B) Phase contrast images for hACE2-HEK cells treated with compound Q34 at concentrations from 0 to 100 μM along with SARS-CoV-2 virus. Scale bar: 50 μm. (C) The percentage of SARS-CoV-2 infected cells in hACE2-HEK cells treated with compound Q34 at concentrations from 0 to 100 μM along with SARS-CoV-2 virus. n = 10 image fields. ∗∗∗p < 0.001 by one-way ANOVA. (D) Number of DAPI-positive cells per fields in hACE2-HEK cells treated with compound Q34 at concentrations from 0 to 100 μM along with SARS-CoV-2 virus. n = 10 image fields. ns means p > 0.05 by one-way ANOVA test. (E) The viral RNA level of SARS-CoV-2 in hACE2-HEK cells treated with compound Q34 or chloroquine (Chlo) along with SARS-CoV-2. n = 3 experimental replicates. ∗∗∗p < 0.001 by one-way ANOVA. (F) The RBD level of SARS-CoV-2 in supernatant of hACE2-HEK cells treated with compound Q34 or chloroquine (Chlo) along with SARS-CoV-2. n = 3 experimental replicates. ∗∗∗p < 0.001 by one-way ANOVA. Error bars are SE of the mean for panels (C) to (F).

Encouraged by the potent inhibitory effect of Q34 on cellular entry by the pseudotyped SARS-CoV-2, we next tested whether Q34 could inhibit the infection of authentic SARS-CoV-2 virus. hACE2-HEK cells were pre-treated with Q34 for 2 h at a concentration range of 0–100 μM and then challenged with the SARS-CoV-2 virus in a BSL3 facility. The cells were then incubated with the SARS-CoV-2 virus along with the compound for 24 h. The viral infection rate in treated cells was determined by immunostaining for the SARS-CoV-2 spike protein. Consistent with the suppressive effect on cellular entry by the pseudotyped SARS-CoV-2, Q34 dramatically inhibited the infection of SARS-CoV-2 virus in hACE2-HEK cells at both 10 μM and 100 μM concentrations, compared to the DMSO treatment control (0 μM Q34) (Figures 4A–4C). Moreover, Q34 treatment dramatically reduced viral RNA level in cells that were pre-treated with the compound followed by SARS-CoV-2 infection, compared to that in cells pre-treated with DMSO followed by SARS-CoV-2 infection (Figure 4E). The reduction of viral RNA level induced by Q34 is comparable to that induced by chloroquine (Figure 4E). Remarkably, while Q34 led to nearly complete blockade of SARS-CoV-2 infection at 100 μM, there was no obvious cytotoxicity, revealed by no obvious change in cellular number and morphology compared to the DMSO treatment control (Figures 4B and 4D). The strong inhibitory effect of Q34 on SARS-CoV-2 viral infection of hACE2-HEK cells was further supported by a decreased level of the SARS-CoV-2 spike protein in the supernatant from cell cultures treated with Q34 compared to that treated with DMSO (Figure 4F). An inhibitory effect by chloroquine was also observed as revealed by the reduced spike protein level in the viral supernatant (Figure 4F). These data together demonstrate that compound Q34 efficiently inhibits SARS-CoV-2 infection of human cells without obvious cytotoxicity.

### Compound Q34 inhibits authentic SAR-CoV-2 infection of human iPSC-derived neurons and astrocytes

**Figure 5:**
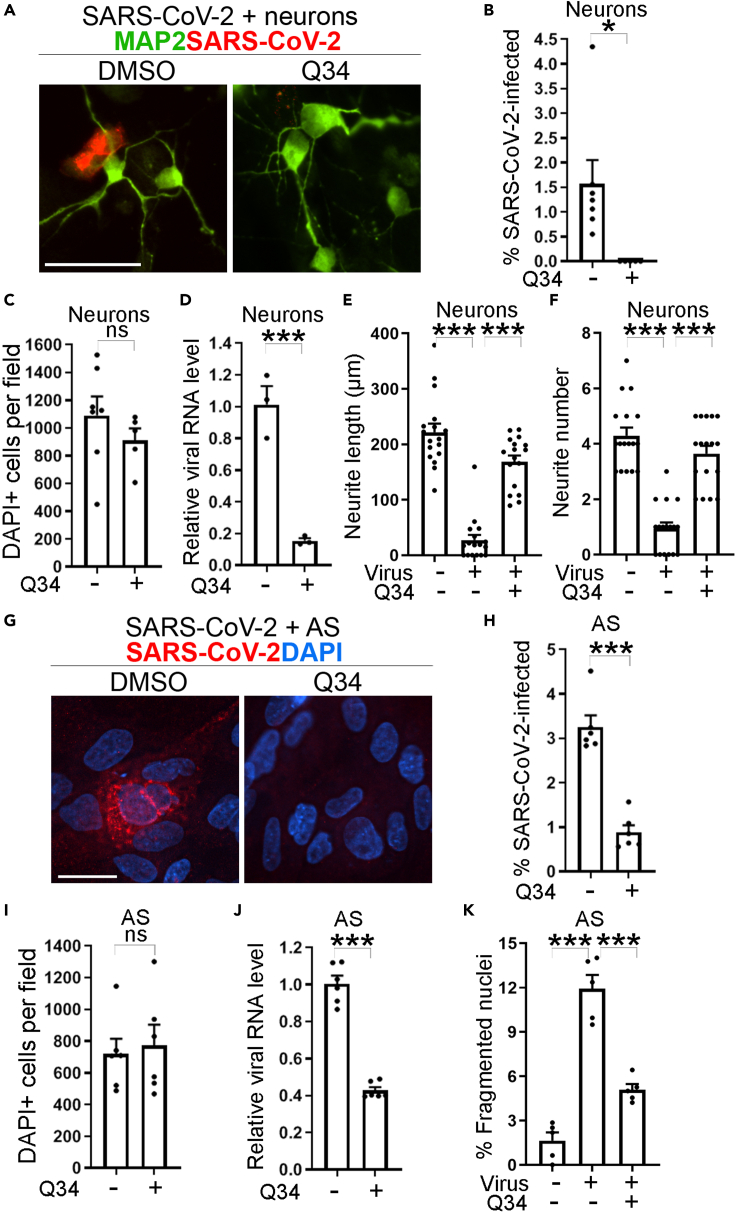
Compound Q34 inhibits authentic SAR-CoV-2 infection of neurons and astrocytes (A and G) Immunostaining for SARS-CoV-2 in neurons (A) or astrocytes (G) treated with compound Q34 along with SARS-CoV-2 virus. Scale bar: 50 μm. (B and H) The percentage of SARS-CoV-2 infected cells in neurons (B) or astrocytes (H) treated with compound Q34 along with SARS-CoV-2 virus. n = 5 image fields. ∗p < 0.05, ∗∗∗p < 0.001 by Student’s t test. (C and I) Number of DAPI-positive cells per fields in neurons (C) or astrocytes (I) treated with compound Q34 along with SARS-CoV-2 virus. n = 5 image fields. ns means p > 0.05 by Student’s t test. (D and J) The viral RNA level of SARS-CoV-2 in neurons (D) or astrocytes (J) treated with compound Q34 along with SARS-CoV-2 virus. n = 3 experimental replicates for D and n = 6 experimental replicates for (J). ∗∗∗p < 0.001 by Student’s t test. (E, F, and K) Compound Q34 treatment alleviated shortened neurite length (E), decreased neurite number (F) in neurons, and increased percentage of fragmented nuclei (K) in astrocytes, resulted from SARS-CoV-2 treatment. n = 17 image fields for (E) and (F). n = 5 image fields for (K). ∗∗∗p < 0.001 by one-way ANOVA test. Error bars are SE of the mean for panels (B)–(F) and (H)–(K).

To test whether Q34 could inhibit the infection of authentic SARS-CoV-2 virus in physiologically relevant human cells without overexpression of hACE2, we developed human iPSC-derived neurons and astrocytes for compound treatment and SARS-CoV-2 challenge as we described previously (Wang et al., 2021). Human iPSC-derived neurons or astrocytes were pre-treated with Q34 for 2 h and then challenged with the SARS-CoV-2 virus in a BSL3 facility. The viral infection rate in treated cells was determined by immunostaining for the SARS-CoV-2 spike protein. Consistent with the suppressive effect on the infection of hACE2-HEK cells by SARS-CoV-2, Q34 dramatically inhibited the infection of SARS-CoV-2 in both neurons (Figures 5A and 5B) and astrocytes (Figures 5G and 5H), compared to the DMSO control. In addition, Q34 treatment dramatically reduced viral RNA levels in neurons and astrocytes, compared to the DMSO control (Figures 5D and 5J). Importantly, Q34 treatment resulted in no obvious cytotoxicity, as revealed by no obvious change in cell number compared to the DMSO control (Figures 5C and 5I). Moreover, Q34 treatment dramatically alleviated the cellular effects resulted from SARS-CoV-2 treatment in both neurons and astrocytes. Q34 treatment largely prevented neurite degeneration, including shortened neurite length (Figure 5E) and decreased neurite number (Figure 5F) in neurons. Treatment with Q34 also rescued cell apoptosis induced by SARS-CoV-2 in astrocytes as revealed by decreased percentage of fragmented nuclei in Q34-treated cells (Figure 5K). These data together demonstrate that Q34 efficiently inhibits SARS-CoV-2 infection of physiologically relevant human cells without obvious cytotoxicity and can prevent pathological consequences resulted from SARS-CoV-2 infection.

### Compound Q34 inhibits authentic SAR-CoV-2 infection of human lung cells

**Figure 6:**
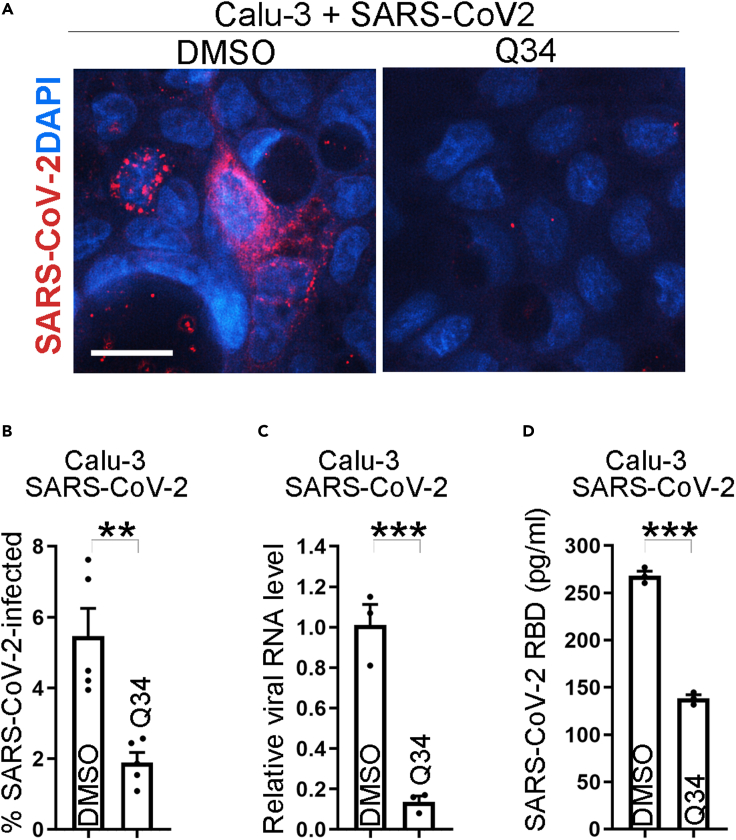
Compound Q34 inhibits authentic SAR-CoV-2 infection of lung cells (A) Immunostaining for SARS-CoV-2 in Calu-3 cells treated with compound Q34 along with SARS-CoV-2 virus. Scale bar: 50 μm. (B) The percentage of SARS-CoV-2 infected cells in Calu-3 cells treated with compound Q34 along with SARS-CoV-2 virus. n = 5 image fields. ∗∗∗p < 0.001 by Student’s t test. (C) The viral RNA level of SARS-CoV-2 in Calu-3 cells treated with compound Q34 along with SARS-CoV-2. n = 3 experimental replicates. ∗∗∗p < 0.001 by Student’s t test. (D) The RBD level of SARS-CoV-2 in supernatant of Calu-3 cells treated with compound Q34 along with SARS-CoV-2. n = 3 experimental replicates. ∗∗∗p < 0.001 by Student’s t test. Error bars are SE of the mean for panels (B) to (D).

To test whether Q34 could inhibit the infection of authentic SARS-CoV-2 virus in human cells from lung tissues that are highly susceptible to SARS-CoV-2 attack, we tested compound treatment in human lung Calu-3 cells challenged with authentic SARS-CoV-2. Calu-3 cells were pre-treated with Q34 for 2 h and then challenged with the SARS-CoV-2 virus in a BSL3 facility. The viral infection rate in treated cells was evaluated by immunostaining for the SARS-CoV-2 spike protein. Consistent with the results from hACE2-HEK cells, iPSC-derived neurons and astrocytes, Q34 inhibited the infection of SARS-CoV-2 in Calu-3 cells potently as revealed by the dramatically reduced percentage of spike+ cells (Figures 6A–6B). Accordingly, the viral RNA level and the spike protein level in the supernatant were dramatically reduced in Calu-3 cells treated with Q34 compared to that treated with the DMSO control (Figures 6C–6D). Taken together, these data demonstrate that Q34 efficiently inhibits SARS-CoV-2 infection of COVID disease-relevant human cells.

## Discussion

The COVID-19 outbreak spreads rapidly around the world without a foreseeable stop. While vaccine has helped to prevent SARS-CoV-2 spread substantially, no drug for SARS-CoV-2 has yet been approved except remdesivir, which has been granted emergency use authorization (EUA) for the treatment of COVID-19. In this study, we screened a compound library containing the NCI-DTP compounds using a luciferase-based pseudotyped SARS-CoV-2 virus-mediated cell entry assay. This screen allowed us to identify a small molecule compound Q34 that can inhibit the cellular entry by the pseudotyped SARS-CoV-2 and cellular infection by the authentic SARS-CoV-2 virus potently. Moreover, there is no obvious cytotoxicity in cells treated with this compound in the same dose range that exhibited potent inhibitory effect on SARS-CoV-2 infection.

We used a pseudovirus-based assay for the screening. Pseudovirus is much safer and easier to handle than the actual SARS-CoV-2 virus because the pseudotyped virus no longer contains the virulent viral components and involves only a single round of replication (Nie et al., 2020; Zhao et al., 2013). Moreover, the luciferase reporter in the pseudoviral system provides a quantitative readout, making it a sensitive and robust screening platform. Because pseudovirus can only undergo one round of infection without the ability to replicate in the target cells (Zhao et al., 2013), suppression of pseudovirus infection can be used as a readout for inhibition of cellular entry by the virus (Tai et al., 2020). Therefore, the luciferase-based pseudovirus assay provides a safe and suitable alternative to authentic SARS-CoV-2 viral assay with BSL3 restrictions to screen viral entry inhibitors.

Vast efforts have been made to develop specific interventions for SARS-CoV-2, in addition to testing general antiviral drugs and therapies for immune regulation to alleviate the symptoms of COVID-19. Various viral components have been used as targets against SARS-CoV-2. Computer-aided virtual screen and structure-based inhibitor development have used multiple viral components as targets, including the spike protein (Wu et al., 2020a), the spike protein RBD (Benitez-Cardoza and Vique-Sanchez, 2020), RNA polymerase (Wu et al., 2020a; Mirza and Froeyen, 2020; Naik et al., 2020), helicase (Mirza and Froeyen, 2020; Naik et al., 2020), endoribonuclease (Naik et al., 2020), exoribonuclease (Naik et al., 2020), methyltransferase (Naik et al., 2020), and proteases, such as the main protease (Jin et al., 2020; Ton et al., 2020; Mirza and Froeyen, 2020; Tsuji, 2020; Gentile et al., 2020; Gurung et al., 2020; Das et al., 2020; Mohammad et al., 2020; Gahlawat et al., 2020; Arun et al., 2020; Peele et al., 2020; Narkhede et al., 2020; Qiao et al., 2021; Naik et al., 2020; Wu et al., 2020a; Chen et al., 2020; Rathnayake et al., 2020), and papain-like protease (PLpro) (Wu et al., 2020a).

In addition to viral components, host proteins are also explored as targets for intervening SARS-CoV-2 infection. A map for SARS-CoV-2 and human protein interaction has been used to identify 66 SARS-CoV-2 interacting host factors targeted by 69 compounds (Gordon et al., 2020). Inhibitors for host transmembrane protease serine 2 (TMPRSS2) (Rahman et al., 2020), an enzyme that facilitates viral particle entry into host cells (Hoffmann et al., 2020), various indirect modulators for ACE2 and cellular molecules regulating ACE2 expression (Ragia and Manolopoulos, 2020), and inhibitors for host cell surface GRP78 (Palmeira et al., 2020), a molecule that is predicted to bind the SARS-CoV-2 spike protein by molecule docking (Ibrahim et al., 2020) have also been identified.

Besides these explorations that started with specific targets of viral components or host proteins, screening for inhibitors for SARS-CoV-2 has been investigated. Large-scale compound repurposing has been done for clinical-stage or FDA-approved small molecules to identify potential SARS-CoV-2 antiviral drugs (Riva et al., 2020), with a pan-coronavirus inhibitor, clofazimine, identified to target spike-mediated cell fusion and viral helicase activity (Yuan et al., 2021). Screening of existing pharmaceuticals and herbal medicines has identified potential molecule inhibitors with anti-infective activity against SARS-CoV-2 (Jan et al., 2021). Screening of kinase inhibitors has identified several inhibitors in clinical trials for cancer treatment that can be potentially repurposed to inhibit SARS-CoV-2 by targeting host-pathogen interaction (Garcia et al., 2021). In this study, we identified compound Q34 that efficiently blocks SARS-CoV-2 entry into human cells by screening NCI-DTP compounds. This small molecule compound can inhibit SARS-CoV-2 infection potently but have no detectable cytotoxicity. This compound and its analogs have great potential to be developed into effective prophylactics and therapeutics for COVID-19 and related pandemics in the future. It would also be important to further explore the value for combinatorial therapies using Q34 compound identified by us and compound inhibitors identified in other studies to enhance clinical efficacy and develop potential therapeutic drugs for COVID-19 and future-related pandemic.

### Limitations of the study

While compound Q34 identified in this study holds promising potential to be developed as an inhibitor of SARS-CoV-2 infection and for COVID-19 disease treatment, there are a few limitations that need to be addressed before moving Q34 or it structural analog(s) forward to the clinic. First, animal studies are needed to test *in vivo* toxicity, efficacy, and pharmacokinetics of Q34 or its structural analogs *in vivo*. Moreover, it would be helpful to test the efficacy of Q34 in inhibiting viral infection of various types of SARS-CoV-2 variants. To broaden the application potential of Q34, it would be interesting to test if Q34 can work synergistically with other therapeutics (e.g. viral replication inhibitors) to inhibit SARS-CoV-2 infection. Mechanistically, this study suggests that compound Q34 may interact with factors different from reported SARS-CoV-2 cellular receptors, such as ACE2, TMPRSS2, and NRP1. It would be important to identify host factor(s) that Q34 interacts with to inhibit SARS-CoV-2 cellular entry in the future.

## STAR★Methods

### Key resources table

**Table:** 

REAGENT or RESOURCE	SOURCE	IDENTIFIER
**Antibodies**		

Goat polyclonal anti-ACE2	R&D SYSTEMS	Cat#AF933; RRID:AB_355722
Chicken polyclonal anti-MAP2	ABCAM	Cat#ab5392; RRID: AB_2138153
Mouse monoclonal anti-GAPDH	SANTA CRUZ	Cat#sc-47724; RRID:AB_627678
Rabbit monoclonal anti-SARS-CoV-2 Spike	Sino biological	Cat#40150-R007; RRID: AB_2827979

**Bacterial and virus strains**		

SARS-CoV-2 (USA-WA1/2020) .	Wang et al.	N/A

**Chemicals, peptides, and recombinant proteins**		

DMEM	Corning	Cat# 15-013-CV
Fetal Bovine Serum	Sigma-Aldrich	Cat# F4135
L-glutamine	Gibco	Cat# 25030-081
Antibiotic-Antimycotic	Gibco	Cat# 15240-062
Minimum Essential Medium	Caisson Labs	Cat# MEL06
GlutaMax	Gibco	Cat# 35050079
NEAA	Thermo Fisher Scientific	Cat# 11140076
Chloroquine	Sigma-Aldrich	Cat # C6628

**Critical commercial assays**		

MycoAlert PLUS Mycoplasma Detection Kit	Lonza	Cat# LT07-318
ONE-Glo Luciferase Assay System	Promega	Cat# E6120
human SARS-CoV-2 RBD ELISA kit	Invitrogen	Cat# EH492RB
Tetro cDNA synthesis Kit	BioLINE	Cat# BIO-65043
DyNAmo ColorFlash SYBR Green qPCR Kit	Thermo Scientific	Cat#F416XL
SARS-CoV-2 Spike S1-Biotin: ACE2 TR-FRET Assay Kit	BPS Bioscienc	Cat#79949-1
Cathepsin B Inhibitor Screening Assay Kit	BPS biological	Cat# 79590
Cathepsin L Inhibitor Screening Assay Kit	BPS biological	Cat# 79591

**Experimental models: cell lines**		

HEK293T	ATCC	Cat#CRL-3216
human iPSC (AG06869)-derived neurons	Wang et al.	N/A
human iPSC (AG06869)-derived astrocytes	Wang et al.	N/A
Calu-3	ATCC	Cat# HTB-55
AG06869 fibroblasts	Coriell	Cat# AG06869

**Oligonucleotides**		

nCoV-N1-F 5′-GAC CCC AAA ATC AGC GAA AT-3′	IDT	N/A
nCoV-N1-R 5′-TCT GGT TAC TGC CAG TTG AAT CTG-3′	IDT	N/A

**Recombinant DNA**		

hACE2	Addgene	#1786
pcDNA3.1-SARS2-Spike	Addgene	#145032
pCSDest-HA-TMPRSS2	Addgene	#154963
MAC-NRP1	Addgene	#158384

**Software and algorithms**		

Graphpad Prism 8	Graphpad Software	RRID: SCR_002798
NIS-Elements AR	Nikon	RRID: SCR_014329

### Resource availability

#### Lead contact

Further information and requests for resources and reagents should be directed to the lead contact, Yanhong Shi (yshi@coh.org).

#### Materials availability

This study did not generate new unique reagents.

### Experimental model and subject details

#### Generation of iPSC

AG06869 fibroblasts (Cat# AG06869) was obtained from Coriell and reprogrammed to pluripotent stem cells as we described previously (Wang et al., 2021; Chen et al., 2021; Feng et al., 2020; Sun et al., 2020). The induced pluripotent stem cells (iPSCs) were used to differentiate into neurons and astrocytes following the protocols we described previously (Wang et al., 2021; Li et al., 2018a).

#### Cell culture

hACE2-HEK293T (hACE2-HEK) cells were obtained by transducing hACE2 expressing lentivirus (hACE2-encoding plasmid from Addgene #1786 was used for cloning hACE2 into a lentiviral vector) into HEK293T cells. HEK293T-luc (HEK-luc) cells were obtained by transducing HEK293T cells with a luciferase reporter-encoding lentivirus pHIV7-eGFP-ffluc. Both hACE2-HEK and HEK-luc cells were cultured in DMEM medium (Corning, Catalog # 15-013-CV) supplemented with 10% Fetal Bovine Serum (FBS) (Sigma, Catalog #F4135), 2 mM L-glutamine (Gibco, Catalog # 25,030-081) and 1 X Antibiotic-Antimycotic (Gibco, Catalog # 15,240-062) at 37°C. Calu-3 cells were cultured in Minimum Essential Medium (Caisson Labs, Catalog # MEL06) supplemented with 10% Fetal Bovine Serum (FBS) (Sigma, Catalog #F4135), 1 X GlutaMAX (Gibco, Catalog # 35,050,079), 1 X MEM NEAA (Gibco, Catalog # 11,140,076) and 1 X Antibiotic-Antimycotic (Gibco, Catalog # 15,240-062) at 37°C. All cultures were confirmed for lack of mycoplasma contamination using MycoAlert PLUS Mycoplasma Detection Kit (Lonza, Catalog # LT07-318).

### Method details

#### Viral preparation

The SARS-CoV-2 pseudovirus was prepared by transfecting the plamids pMDL, pREV, pcDNA3.1-SARS2-Spike (Addgene #145032), and pHIV7-eGFP-ffLuc into HEK293T cells by calcium phosphate precipitation. Virus containing medium was collected 3 days after transfection. The VSV-G pseudovirus was prepared by transfecting the plamids pMDL, pREV, pVSV-G, and pHIV7-eGFP-ffLuc into HEK293T cells by calcium phosphate precipitation as we described previously (Cui et al., 2016, 2017). Virus containing medium was collected 3 days after transfection.

#### Library screen by reporter assay

hACE2-HEK cells were seeded in 48-well plates at 10,000 cells per well one day before compound treatment. Cells were pre-treated with the vehicle control DMSO or 10 μM compounds for 2 h followed by addition of the SARS-CoV-2 pseudovirus. Cells were incubated with the SARS-CoV-2 pseudovirus along with DMSO or individual compounds for 24 h, then subjected to medium change. Chloroquine (Sigma, Catalog #C6628) was included as control. For antibody treatment, 20 μg/mL anti-Spike antibody (Sino biological, Catalog # 40,150-R007) was used. Luciferase activity was measured 3 days after virus treatment using the ONE-Glo Luciferase Assay System (Promega, Catalog #E6120).

#### Compound validation by reporter assay

hACE2-HEK cells were seeded in 48-well plates at 10,000 cells per well one day before compound treatment. Cells were pre-treated with DMSO or specific compounds at the indicated dose for 2 h, followed by addition of the SARS-CoV-2 pseudovirus or the VSV-G pseudovirus for 2 h or 24 h, then subjected to medium change. Luciferase activity was measured 3 days after virus treatment using the ONE-Glo Luciferase Assay System (Promega, Catalog #E6120).

#### Compound toxicity test

HEK-luc cells were seeded in 48-well plates at 10,000 cells one day before compound treatment. Cells were treated with DMSO or specific compounds at the indicated doses for 4 h (2 h + 2 h) or 26 h (2 h + 24 h) followed by medium change. Luciferase activity was measured 3 days after compound treatment using the ONE-Glo Luciferase Assay System (Promega, Catalog #E6120).

#### Inhibition of SARS-CoV-2 infection by the test compound

hACE2-HEK cells, human iPSC-derived neurons or astrocytes, Calu-3 cells were seeded in 96-well plates at 20,000 cells per well one day before compound treatment. Cells were pre-treated with DMSO or specific compounds for 2 h followed by addition of the SARS-CoV-2 virus strain (Wang et al., 2021) at MOI of 0.1 (hACE2-HEK and Calu-3) or MOI of 1 (neuron and astrocyte). Cells were incubated with the SARS-CoV-2 virus along with DMSO or specific compound for 24 h–48 h (hACE2-HEK, Calu-3 and astrocyte) or 72 h (neuron). At the end of the treatment, the cell culture supernatant were harvested with viral RBD level tested by human SARS-CoV-2 RBD ELISA kit (Invitrogen, Catalog # EH492RB), cells were harvested in Trizol for RNA extraction or fixed with 4% PFA, followed by immunostaining using antibody specific for the SARS-CoV-2 spike protein (1:200, Sino biological, Catalog # 40,150-R007).

#### RT-PCR

Total RNA was isolated using Trizol reagent (Ambion) and subjected to reverse transcription performed using the Tetro cDNA synthesis Kit (BioLINE). RT-PCR reactions were performed using SYBR Green Master Mix (Thermo Scientific) on the Step One Plus Real-Time PCR instrument (Applied Biosystems). The following primers were used for RT-PCR: nCoV-N1-F 5′-GAC CCC AAA ATC AGC GAA AT-3′; and nCoV-N1-R 5′-TCT GGT TAC TGC CAG TTG AAT CTG-3′; and ACTIN F, 5′-CCG CAA AGA CCT GTA CGC CAA C-3′; and ACTIN R, 5′-CCA GGG CAG TGA TCT CCT TCT G-3′. ACTIN was used as the reference gene for normalization. The ΔΔCt method was used for quantification analysis.

#### TR-FRET and cathepsin B/L activity assay

To test ACE2 and SARS-CoV-2 Spike interaction, the SARS-CoV-2 Spike S1-Biotin: ACE2 TR-FRET Assay Kit (BPS Bioscience, Catalog #79949-1) was used by including 1 mM compound Q34 or 10 μg/mL anti-Spike antibody (Sino biological, Catalog # 40,150-R007). To test the Cathepsin B/L activity, Cathepsin B Inhibitor Screening Assay Kit (BPS biological, Catalog # 79,590) and Cathepsin L Inhibitor Screening Assay Kit (BPS biological, Catalog # 79,591) were used by including 100 μM compound Q34 or 0.1 μM E64 provided in the kits.

### Quantification and statistical analysis

GraphPad Prism 8 with default setting was used for statistical analyses. The Student's t-test was used for statistical significance test when two groups of samples were compared. The one-way ANOVA test was used for statistical significance test when more than two groups of samples were compared. Values were presented as ∗p < 0.05, ∗∗p < 0.01, ∗∗∗p < 0.001. Error bars are s.e. of the mean. All statistical details of experiments can be found in figure legends.

## Data Availability

All data reported in this paper will be shared by the lead contact upon request. This paper does not report original code. Any additional information required to reanalyze the data reported in this paper is available from the lead contact upon reasonable request.
