# Supplementary material for: Compound screen identifies the small molecule Q34 as an inhibitor of SARS-CoV-2 infection
Source: iScience. 2021 Dec 24;25(1):103684. doi: 10.1016/j.isci.2021.103684 (PMC8704726; doi:10.1016/j.isci.2021.103684)
Supplement: Document S1. Figures S1 and S2 [file mmc1.pdf]

iScience, Volume *25*

Supplemental information

Compound screen identifies the small molecule Q34 as an inhibitor of SARS-CoV-2 infection

Qi Cui, Gustavo Garcia Jr., Mingzi Zhang, Cheng Wang, Hongzhi Li, Tao Zhou, Guihua Sun, Vaithilingaraja Arumugaswami, and Yanhong Shi

Supplementary Figures and Legends

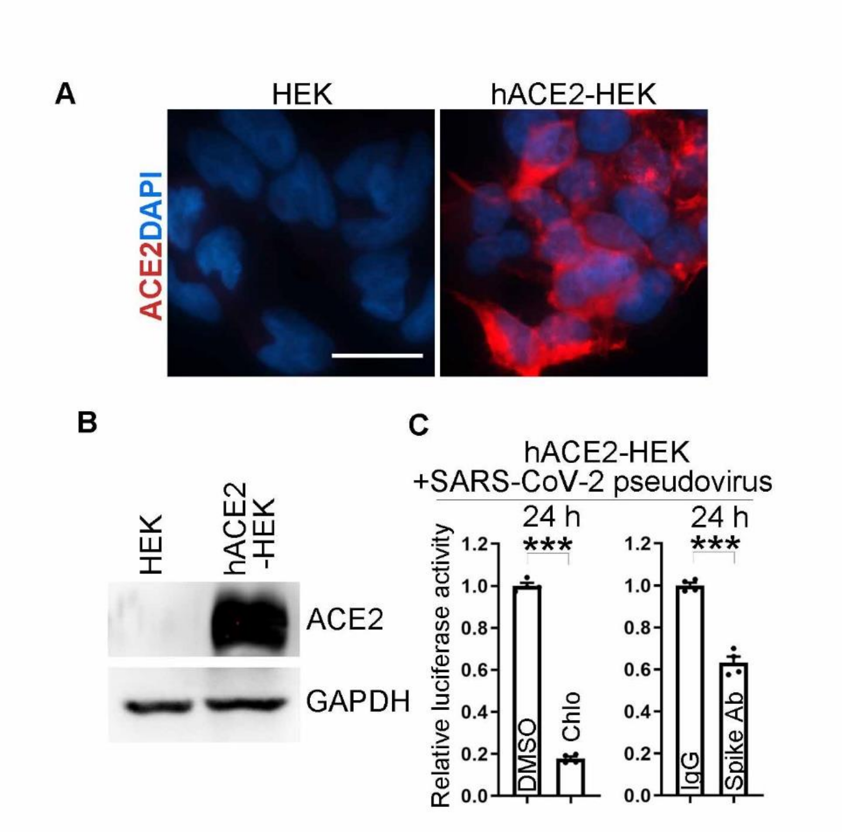

Figure S1. Luciferase reporter system for pseudotyped SARS-CoV-2 cellular entry test in hACE2-HEK cells, Related to Figure 1. (A) Immunostaining for ACE2 in HEK and hACE2-HEK cells. Scale bar: 50 µm. (B) Western blot analysis for ACE2 in HEK and hACE2-HEK cells. (C) Compound chloroquine (chlo) or anti-Spike antibody (spike Ab) inhibits pseudotyped SARS-CoV-2 entry into hACE2-HEK cells. n=4 experimental replicates. ***$p < 0.001$ by Student's t test. Error bars are SE of the mean.

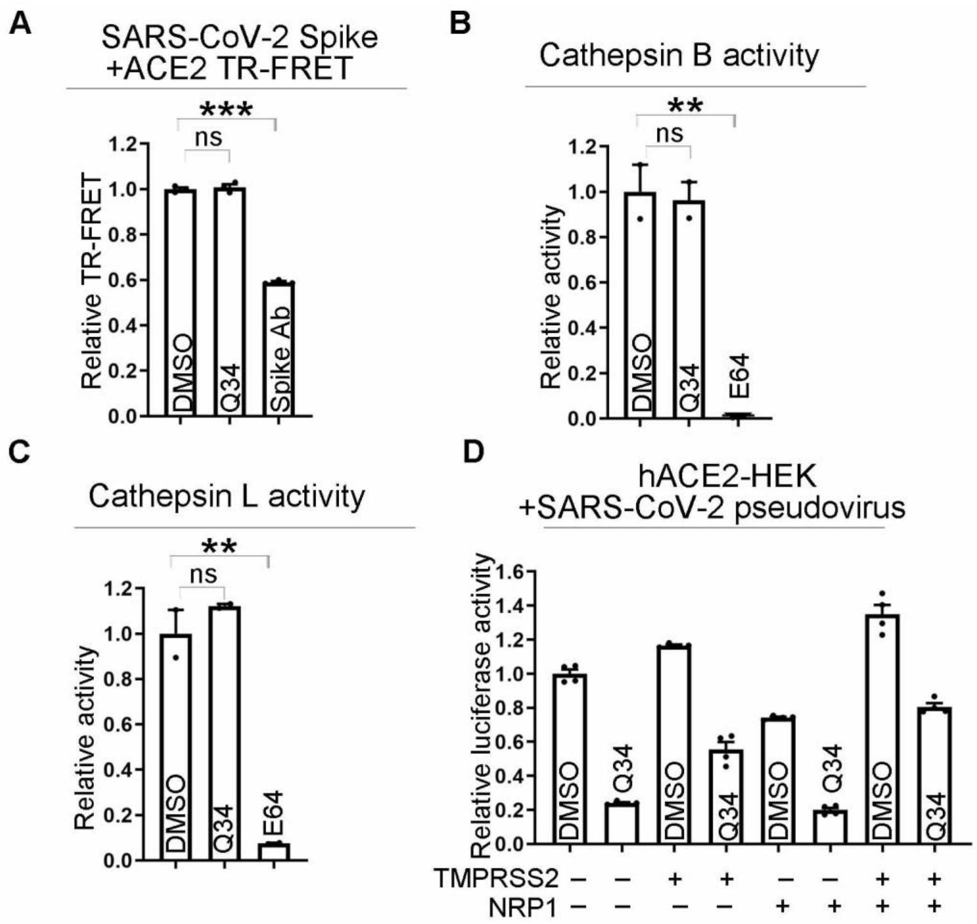

Figure S2. Compound Q34 inhibits cellular entry of pseudotyped SARS-CoV-2, Related to Figure 2. (A) Time-resolved fluorescence energy transfer (TR-FRET) test for ACE2 and SARS-CoV-2 Spike protein interaction in the presence of compound Q34 or anti-Spike antibody (spike Ab). ns means $p > 0.05$ and ***$p < 0.001$ by one-way ANOVA test. (B) The activity of cathepsin B in the presence of compound Q34 or E64. ns means $p > 0.05$ and **$p < 0.01$ by one-way ANOVA test. (C) The activity of cathepsin L in the presence of compound Q34 or E64. ns means $p > 0.05$ and **$p < 0.01$ by one-way ANOVA test. (D) TMPRSS2 and NRP1 increase pseudotyped SARS-CoV-2 entry into hACE2-HEK cells with or without treatment of compound Q34. n=4 experimental replicates. Error bars are SE of the mean.
